# Can transrectal ultrasonography distinguish anorectal malignant melanoma from low rectal adenocarcinoma? A retrospective paired study for ten years

**DOI:** 10.1186/s12876-022-02237-6

**Published:** 2022-04-05

**Authors:** Jingwen Yan, Jigang Jing, Shuang Wu, Lacong Geiru, Hua Zhuang

**Affiliations:** Department of Diagnostic Ultrasound, West China Hospital, Sichuan University, No. 37 Guoxue Road, Wuhou District, Chengdu, 610041 Sichuan Province China

**Keywords:** Diagnosis, Anorectal malignant melanoma, Low rectal adenocarcinoma, Transrectal ultrasonography

## Abstract

**Background:**

Anorectal malignant melanoma (ARMM) and low rectal adenocarcinoma (LRAC) have some similarities in clinical behaviors, histopathological characteristics and ultrasonographic findings, diagnostic errors are common. By comparing the transrectally ultrasonographic features between the two tumors, we propose to provide more possibilities in differentiating them.

**Methods:**

The data of 9 ARMMs and 27 age- and gender-matched LRACs (the lower margin below the peritoneal reflection) in West China Hospital Sichuan University between April 2008 and July 2019 were retrospectively reviewed. The ultrasonic features between the two groups were compared.

**Results:**

Transrectal ultrasonography (TRUS) showed that the length of ARMM was shorter than that of LRAC (28.22 ± 12.29 mm vs. 40.22 ± 15.16 mm), and ARMM had a lower position than that of LRAC (the distance to anal verge was 50.78 ± 11.70 vs. 63.81 ± 18.73 mm). Unlike LRAC, the majority of ARMM in our study was confined to the intestinal mucosa/submucosa (66.67/25.93%) (*P* < 0.05).

**Conclusions:**

Based on the data of our study, several ultrasonographic findings (length, invasion depth, and position) of ARMM were significantly different from LRAC. Accordingly, more attention should be paid to masses at anorectal junction with lower position, shorter length, and shallower infiltration depth. Instead of the most common tumor, LRAC, ARMM should be taken into account to avoid a misdiagnosis, which will result in a poorer prognosis.

## Background

Primary anorectal malignant melanoma (ARMM) is a rare disease, with an annual incidence less than 0.3 per million in the USA [1]. The prevalence has been increasing from 6.99% in 2004 to 10.53% in 2015 [2]. Owing to its non-specific symptoms and clinical signs, early diagnosis of ARMM is very difficult, and it is inclined to be misdiagnosed as anorectal cancer or hemorrhoids. It was reported that the misdiagnosis rate could be up to 80% (mainly as anorectal cancer) [3]. Adenocarcinoma is the most common type of low rectal malignant tumors. Approximately 30% of ARMM is amelanotic and can endoscopically resemble poorly differentiated adenocarcinoma, contributing to the difficulty in diagnosis [4]. Although there are some similarities between ARMM and low rectal adenocarcinoma (LRAC), their prognosis and treatments are different. ARMM is more aggressive. At the time of diagnosis, 61% of patients with ARMM already have distant metastases, leading to a poor prognosis, with a median post-treatment survival time of 12–20 months and a 5-year survival rate of 6–22% [3, 5–10]. So, it is extremely imperative to reduce the misdiagnosis rate as well as the delay in timely treatments.

At present, transrectal ultrasonography (TRUS) is an important imaging method for preoperative evaluation of anorectal diseases. As most commonly malignant anorectal tumors are LRACs, the ultrasonographic manifestations reported in the literature have mainly been focused on rectal cancer. There has been no study reported on the differential diagnosis of ARMM and LRAC based on ultrasonographic features. We retrospectively collected and analyzed the data in our hospital, trying to provide the possibility of differentiating ARMM from LRAC by comparing the transrectally ultrasonic features of both tumors.

## Methods

### Patients

This retrospective study was approved by the Ethics Committee of West China Hospital, Sichuan University. Written informed consents were obtained from all patients. From April 2008 to July 2019**,** the date of 9 patients with ARMM who had undergone TRUS scanning before treatments were collected in our institution. Seven women and 2 men were included. The age of the 9 patients ranged from 59 to 69 years (65.33 ± 3.23 years). All patients underwent colonoscopy and biopsy, of whom four lesions were black and were diagnosed as ARMM. Two were suggested colorectal cancer due to the lack of tumor pigmentation. And the remaining three cases were diagnosed as colorectal cancer, but the color of these lesions were not recorded. The initial misdiagnosis rate reached 55.56% before postoperative pathological diagnosis and immunohistochemistry analysis. In our database during the corresponding period, 27 gender- and age-matched cases pathologically diagnosed with LRAC were randomly selected, every patient of this group also underwent colonoscopy and biopsy, all were suggested adenocarcinoma.

### Ultrasound evaluation

TRUS was performed by MyLab Twice (Esaote, Genova, Italy) or Hivision Preirus (HITACHI, Tokyo, Japan) equipped with a biplane endoscopic probe (TRT33, linear frequency of 4–13 MHz, convex frequency of 3–9 MHz; EUP-R54AW-33, linear frequency of 4-15 MHz, convex frequency of 3-9 MHz, respectively), which was operated by two sonographers with at least 5 years of experience and were randomly assigned per the hospital's daily schedule. We retrospectively analyzed all the 36 TRUS reports from our institution of the two groups. The ultrasound images were reviewed to record the lesion location, length, echotexture (homogeneous/heterogeneous), thickness, depth of invasion (DOI), the distance from the midpoint/the inferior border of the tumor to the anal verge along the long axis (M-Dist /I-Dist), the presence of lymph node metastasis, the size of these lymph nodes, blood flow signal, peak systolic velocity (PSV), and resistance index (RI).

According to the location of the tumor midpoint along the short axis of tumor, the patients were divided into the anterior wall group (A-group, lithotomy position, 10–2 o'clock) and non-anterior-group (NA-group, lithotomy position, other locations). On the basis of the DOI, patients were divided into shallow (mucosa-submucosa) group and deep (muscularis propria-adventitia) group. Meanwhile, we use the M-dist to compare the position of tumor (low/high).

### Statistical analysis

Statistical package for social analysis (SPSS for Windows, IBM Corp, USA) version 25.0 was used for data analysis. Data were presented as mean ± standard deviation (SD), frequency, and percent. For statistical analysis, we used Student's t-test for continuous variables, and two-tailed Fisher’s exact test for categorical variables. *P *values < 0.05 are considered statistically significant for all tests.

## Results

**Fig. 1:**
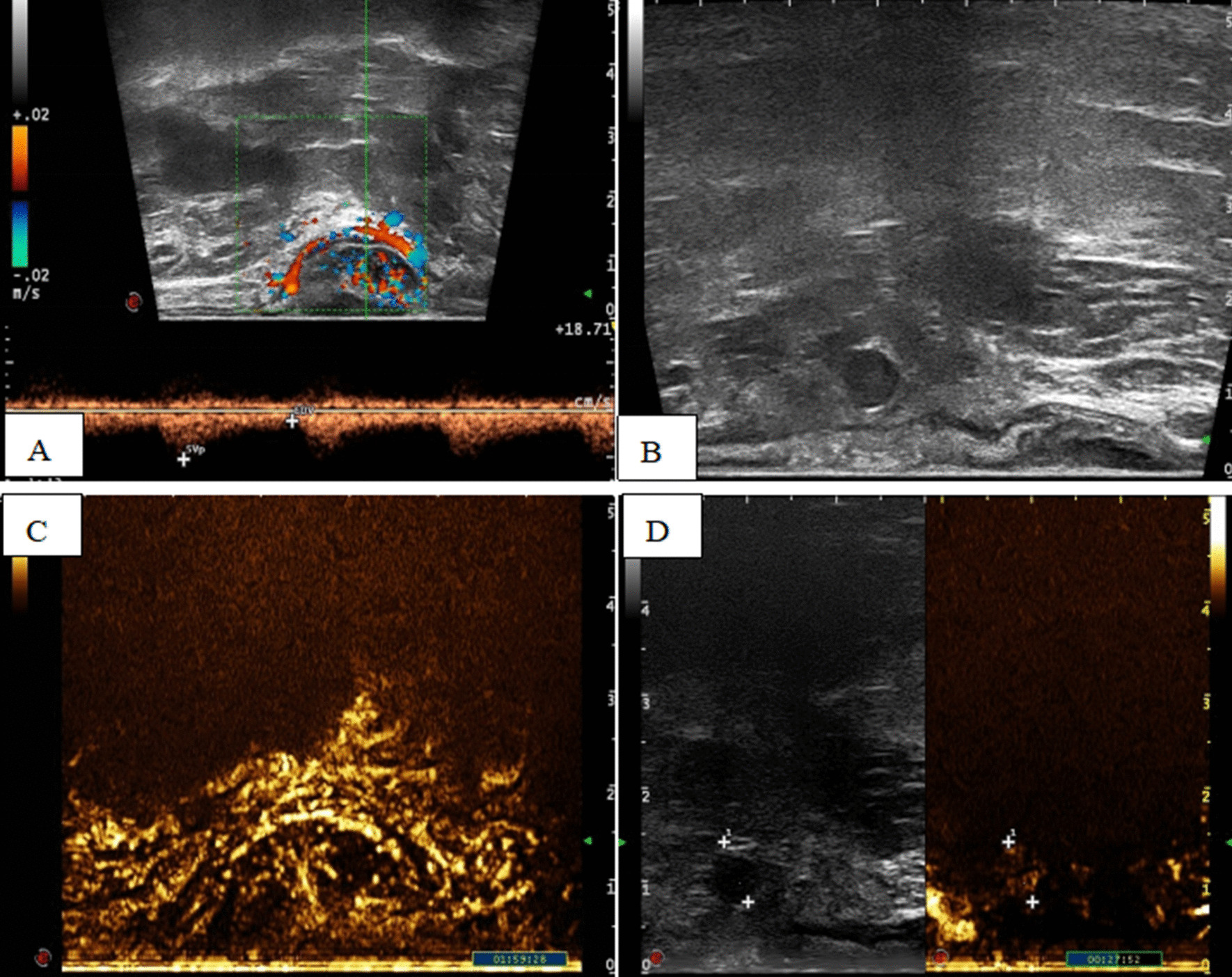
The ultrasonic image of an ARMM (Anorectal malignant melanoma). A 67-year-old female patient. **A** Rich blood flow signals at the marginal and inner part of the mass, the arterial pulse spectrum was detected, and PSV was 14.3 cm/s, RI was 0.82. **B** The presence of perirectal lymph node metastasis, and the maximum diameter of the node was 7 mm. **C** The mass was completely cleared at 1 min and 59 s after contrast-enhanced ultrasonography (CEUS). **D** Perirectal lymph node showed rapid enhancement and clearance in CEUS. Immunohistochemistry analysis and pathology after surgery: S100 (+), HMB45 (+), CD63 (+), PCK (−), EMA (−), PDL1 (−). TRUS found that the lesion infiltrated the submucosa, while pathology suggested that the tumor was confined to the mucosa

**Fig. 2:**
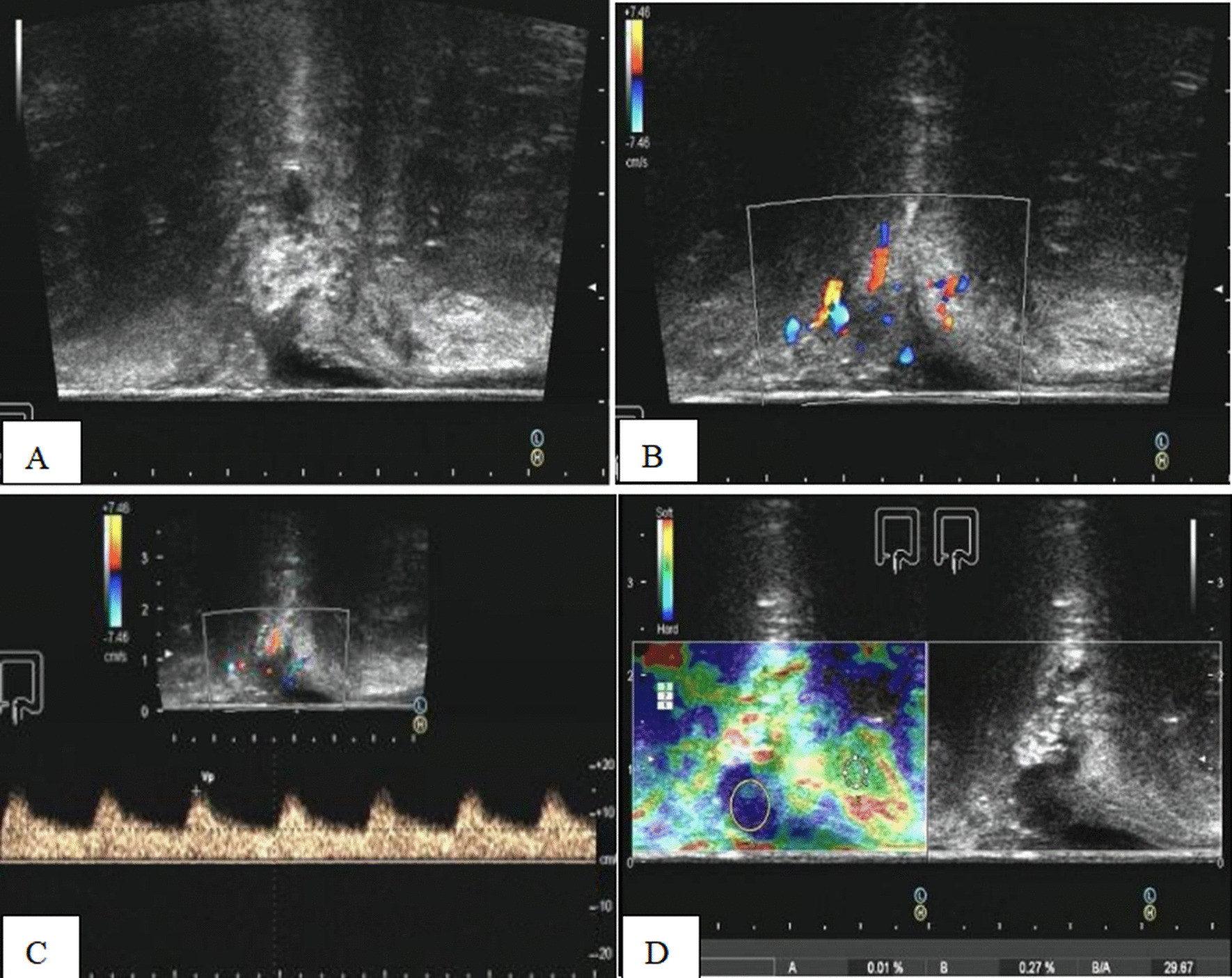
The ultrasonic image of an ARMM. A 61-year-old female patient. **A** A hypoechoic nodule of rectal wall was observed. **B** Punctate blood flow signals were detected in the nodule. **C** LOW resistance arterial spectrum was detected, and PSV was 14.9 cm/s, RI was 0.56. **D** The real-time elastography and strain ratio of the tissue around the intestinal wall to the lesion was 29.67. Immunohistochemistry analysis and pathology after surgery: HMB45 (+), S100 (+), PCK (−), MART1 (+), KI67 45%. Both ultrasound and pathology showed that the DOI was mucosa

According to the TRUS reports of the 36 patients, the mean value of the length of ARMM was shorter than that of LRAC (*P* = 0.037). Compared with LRAC, the position of ARMM tended to be lower, and the DOI of ARMM is shallower (*P* = 0.028, 0.046). Meanwhile, four and 16 patients had perirectal lymph node metastasis in ARMM and LRAC group. We found a total of 7 cases and 24 cases that were detected arterial pulse spectrum in ARMM and LRAC group. However, there were no significant differences in thickness, echotexture, location, PSV, RI, the presence of lymph node metastasis and the size of these lymph nodes. A summary of these results is shown in Table 1. Figures 1 and 2 depict the typical ultrasonic manifestations of ARMM.

**Table 1:** TRUS features of ARMM and LRAC

Features	ARMM (*n* = 9)	LRAC (*n* = 27)	*P*
L (mean ± SD, mm)	28.22 ± 12.29	40.22 ± 15.16	**0.037**
T (mean ± SD, mm)	14.63 ± 6.73	15.75 ± 7.33	0.699
M-Dist (mean ± SD, mm)	50.78 ± 11.70	63.81 ± 18.73	**0.028**
I-Dist (mean ± SD, mm)	36.67 ± 8.82	43.44 ± 19.60	0.181
DOI *n* (%)			**0.046**
Shallow	6(66.67)	7 (25.93)	
Deep	3(33.33)	20 (74.07)	
PSV (cm/s)	31.40 ± 25.56	34.3 ± 22.91	0.547
RI	0.77 ± 0.14	0.83 ± 0.13	0.416
Location *n* (%)			0.255
A	6(66.67)	11(40.74)	
NA	3(33.33)	16 (59.26)	
Echotexture, *n* (%)			0.148
Homogeneous	7(77.78)	26(96.30)	
Heterogeneous	2 (22.22)	1(3.70)	
LN metastasis, *n* (%)	4(44.44)	16(59.26)	0.470
Maximum diameter of LN (mm)	10.25 ± 6.80	6.25 ± 1.75	0.384

TRUS: Transrectal ultrasonography. ARMM: Anorectal malignant melanoma. LRAC: Low rectal adenocarcinoma. L: length. T: Thickness. M-Distance: the distance between the tumor midpoint and the anal verge. I-Distance: the distance between the inferior border of tumor and the anal verge. DOI: depth of invasion. Shallow: mucosa-submucosa invasion. Deep: muscularis propria-adventitia invasion. LN metastasis: the presence of lymph node metastasis. Maximum diameter of LN: the maximum diameter of metastatic lymph node. SD: Standard deviation. Bold indicates statistical significance

## Discussion

Primary malignant melanoma, with up to 1–2% of all malignant tumors, may occur on various sites of the body. Anorectum is the third most common location after skin and retina [11], accounting for 1% of melanomas and 0.5% of anorectal malignancies [12], which was first reported in 1857 by Moore [13]. Although over 150 years have passed, there is not much evidence for standard diagnostic and therapeutic methods due to its rarity [14]. Primary symptoms of ARMM frequently include rectal bleeding, asymptomatic local mass and altered defecation habits, these non-specific clinical manifestations may lead to a delay in diagnosis. Time from symptom onset to disease diagnosis can range from 1 to 24 months [3, 15–17], causing most patients present with advanced disease. Rectal cancer is the third digestive cancer, and LRAC accounts for about 70% of it [18–21]. Both are masses occurring in the anorectum, which share hemafecia as the most common clinical symptom, but the prognosis of rectal cancer is much better than that of ARMM, and the 5-year survival rate is approximately 53% for all stages [22].

The optimal treatment for ARMM is still debated, but surgery is still the main method. The role of adjuvant chemotherapy and immunotherapy in the treatment has not been determined. Traditionally, abdominoperineal resection was considered as the standard treatment of ARMM. In recent years, wide local excision has been performed for localized tumors with similar overall survival rate. A systematic review of the literature showed no significant statistical difference in median survival after these two surgical managements. Wide local excision can preserve the function of anal sphincter, hence, it is recommended whenever possible as an initial, more limited treatment. Whereas the rate of recurrences seems to be lower in patients undergoing abdomioperineal resection [6, 23–25].

In our research, the clinical characteristics of patients with ARMM included: a mean age of 65.33 years (range, 59–69 years), hemafecia as the most common initial symptom (9/9), less males than females affected (a gender ratio of 1:3.5, male to female), namely a female predominance. Meanwhile, patients presented on average 5.4 months after onset of symptoms (range, 0.25–12 months), which were consistent with previous reports [3, 7, 15, 16, 26–30]. Considering the lymphatic drainage of the anorectal region, lymphatic metastases can be usually seen in the inguinal, mesenteric, hypogastric and para-aortic lymph nodes. Distant metastases often appear in the liver, lung, brain and bone [31, 32]. In our study, for the 5 patients with complete follow-up data of ARMM, three of them had metastases during the five years, including liver, liver and inguinal lymph nodes, as well as neck lymph nodes metastases. In the LRAC group, there were 7 occurring distant metastases in 20 cases, including three cases with liver and lung metastases, two with lung metastasis, one with bone metastasis, and one with liver metastasis within five years.

The importance of endoscopy and biopsy in visualizing and sampling ARMM are obvious. ARMM is easily diagnosed if melanin pigment is present. The lesion usually appears as black or brown so that it can be diagnosed by conventional histochemical staining; However, amelanotic melanoma, which is a rare subtype of malignant melanoma, is prone to be mistaken for carcinoma or sarcoma because of the lack of pigmentation. Immuno-staining for Human Melanoma Black-45, Soluble 100% and vimentin are of invaluable help to make an accurate diagnosis [4]. The retrospective study we designed tried to use TRUS to add more information to help in distinguishment between ARMM and LRAC, especially in the difficult cases. Regarding our ultrasonography findings, three significant differences were observed, including length and DOI of tumor, as well as position of tumor.

According to previous studies, ARMM is neuroectodermal neoplasms originating from the melanoblastic cells of the mucosal surface [32, 33]. Most reports have described that ARMM frequently occurs near the dentate line. (52–92% cases) [3, 7, 15, 34]. Our retrospective study confirmed that I-Dist was less than 5 cm in all the 9 patients (36.67 ± 6.82 mm), which was basically consistent with the previously reported cases. However, in most related studies, I-Dist was < 3 cm in the majority of ARMM cases, while in our study, which only occurred in one third of the patients, differing from previous works [35, 36].

Zhang et al. [35] enrolled 216 cases of ARMM, summarized the characteristics of these patients and described that the diameter of the tumor was relatively small (the average tumor size was 33 ± 21 mm). Other literature has reported that the median tumor size at initial presentation is between 20 and 50 mm, and Quan found that the size of lesions were < 10 mm in 25% of cases [6, 7, 26, 37, 38]. Meanwhile, there are some reports describing the mean diameter is relatively large of rectal cancer. Feng et al. [39] reported that the leision diameter of 699 rectal cancer was 44.2 ± 17.3 mm. In our study, the mean length of ARMM (28.22 ± 12.29 mm) was shorter than that of LRAC (40.22 ± 15.16 mm) ultrasonically, which was statistically significant, the reason for it could be that most of the lesions in ARMM showed nodular or massive thickening of the intestinal wall with relatively regular shape, while most of the rectal cancer usually surrounds the intestinal wall and grows irregularly in the depth and length.

The staging of ARMM is different from the cutaneous melanoma. Most researches used the clinical staging, stage I tumors represent localized disease only, stage II regional lymph node involvement and stage III distant metastases [16, 40, 41]. This type of staging system does not take the DOI of tumor into consideration. Some publications have suggested a poorer prognosis with increasing DOI, especially in the muscular layer [36, 42, 43]. According to previous reports, only 11% to 18% of ARMM are confined to the mucosa/anal epithelium [36, 44], between 11 and 29% infiltrate into the submucosa, and about 79% invade the muscularis. It was shown that 44% patients of ARMM already have lymph node metastasis when the tumor infiltrate into the submucosa [43]. In our study, according to pathological verification after surgery, three cases with ARMM were confined to the mucosa, three cases were limited to the submucosa, one case invaded muscularis propria, and two cases extended to the adventitia layer. Compared with postoperative pathology, the accuracy rate of TRUS in assessing the DOI was 77.8%. DOI of the 9 cases of ARMM in this article was relatively shallow compared to the above reports. None of the five patients of ARMM with complete date showed lymph node metastasis in other parts of the body (except for perirectal lymph node) and distant metastasis verified by the clinical, radiological assessment at the time of surgery, which may be due to the earlier consultation and diagnosis time. Most of the LRAC in our study broke through the submucosa and invaded the muscularis propria and adventitia layer (74.07%) ultrasonically, which only accounted for 22.22% in ARMM group. It may help us differentiate between these two diseases.

From our results, it is tough to distinguish ARMM and LRAC by a single feature on TRUS, but adding multiple findings may help in differentiation. According to some published literature, magnetic resonance imaging (MRI) also plays a role in the differential diagnosis of LRAC and ARMM, LRAC is characterized by thickening of the intestinal wall, an eccentrically lobulated tumor in the intestinal lumen, and the tumor usually manifests as iso/hyperintense on T1-weighted images and slightly hypointense on T2-weighted images, while the ARMM usually appears as a bulky intraluminal polypoid mass in the anorectum without colonic obstruction, and the mass shows hyperintense on T1-weighted images and hypointense on T2-weighted images due to the paramagnetic contribution from melanin, however, these features may be absent in patients who have amelanotic melanoma, which creates difficulties in the differential diagnosis of ARMM and LRAC [45–47]. Our study included the cases of a long time because the rarity of ARMM with a very small sample size, moreover, some patients are unable to have an MRI examination, making the contrast-enhanced ultrasonography (CEUS) and MRI results unavailable, so no significant differences were detected in other findings, which can be further studied in larger samples in the future.

Although Computed tomography, MRI, positron emission tomography and endoscopic ultrasound can be used to assess the location, size and DOI of anorectal masses, TRUS is convenient, fast, real-time, anesthesia-free, dynamic and cheap, thus it has been widely used in the examination of anorectal space-occupying lesions. Furthermore, the lower position and smaller size of ARMM make it unusual to be obstructing [48], especially suitable for TRUS.

## Conclusions

In summary, TRUS provides necessary information for the initial assessment of anorectal masses, which could be useful for differential diagnosis on ARMM from the most ordinary anorectal malignancy, LRAC before surgery. Accordingly, compared with LRAC, most ARMM might manifest as relatively short length, low position and shallow DOI ultrasonically. 

## Data Availability

The datasets used and/or analysed during the current study are available from the corresponding author on reasonable request. Because our study involved patients with anorectal melanoma, a rare disease with only 9 cases in 10 years, all of their data were included in our hospital's database, and for keeping track of their disease progression, these patients' names, contact information and addresses have not been blurred in our database. The data of these patients are also valuable information for our hospital, so we didn't choose to make all of these data public in the Data Availability Statement, but if scholars need our data, they can contact our corresponding author, we will be pleased to provide the complete data to help patients with this disease.

## Abbreviations

ARMM: Anorectal malignant melanoma
LRAC: Low rectal adenocarcinoma
TRUS: Transrectal ultrasonography
DOI: Depth of invasion
PSV: Peak systolic velocity
RI: Resistance index
M-Dist: The distance between the tumor midpoint and the anal verge
I-Dist: The distance between the inferior border of tumor and the anal verge
CEUS: Contrast-enhanced ultrasound
MRI: Magnetic resonance imaging
